# Prevalence of hypothyroidism in Japanese chronic kidney disease patients

**DOI:** 10.1080/0886022X.2020.1777162

**Published:** 2020-06-22

**Authors:** Rena Yuasa, Yasushi Ohashi, Akinobu Saito, Kumiko Tsuboi, Seiichiro Shishido, Ken Sakai

**Affiliations:** a Department of Nephrology, Toho University School of Medicine, Tokyo, Japan; b Department of Internal Medicine, Division of Diabetes Metabolism and Endocrinology, Toho University School of Medicine, Tokyo, Japan

**Keywords:** CKD, overt hypothyroidism, subclinical hypothyroidism, non-thyroidal illness, urinary protein

## Abstract

**Background:**

Major symptoms of progressive chronic kidney disease (CKD) are similar to those of hypothyroidism. Hidden symptoms of hypothyroidism underlying CKD are often observed in clinical practice. This study aimed to ascertain the frequency of hypothyroidism complicated by CKD, and to analyze factors impacting thyroid function.

**Methods:**

During the period from April 2012 through October 2016, 510 CKD patients at our outpatient clinic were measured thyroid and kidney function for diagnosing hypothyroidism (overt hypothyroidism, OH; subclinical hypothyroidism, SH; non-thyroidal illness, NTI) and evaluating the stage of CKD. All patients were over 15 years of age.

**Results:**

There were significant differences in age, estimated glomerular filtration rate (eGFR), urinary protein (UP), and serum albumin (Alb) among patients with OH, SH, and NTI compared to the normal group in univariate and multivariate analyses. UP showed the highest odds ratio of OH, SH, and NTI but no differences were recognized in gender in each group. Frequency distribution showed that the prevalence of thyroid dysfunction was greater among more severe stage of CKD with higher amount of UP. OH and SH did not show high positive ratio of anti-thyroglobulin antibody (TgAb) and anti-thyroid peroxidase antibody (TPOAb). NTI and normal subjects showed higher positive ratio as 50.0% and 42.9% of TgAb and TPOAb than OH and SH.

**Conclusions:**

Hypothyroidism complicated by CKD exhibited a high prevalence. Age, eGFR, UP, and serum Alb were related to the prevalence of hypothyroidism, whereas gender was not and this was contradicted to the prevalence of hypothyroidism in general population. The prevalence of OH and SH was higher among patients with higher stage of CKD with increased UP. Hypothyroidism complicated by CKD may involve different onset mechanisms unrelated to antithyroid antibodies (ATAb). In CKD patients, assessments of OH and SH, as well as NTI, are needed for proper diagnosis.

## Introduction

Major symptoms of progressive chronic kidney disease (CKD) are indefinite complaints as general fatigue, edema, shortness of breath, and dizziness. However, similar symptoms are present in hypothyroid patients [1]. Thus, hypothyroidism underlying CKD might be unnoticed by clinicians, although such disease may require treatment.

Multiple groups have investigated the interaction between CKD and hypothyroidism [2–6], but no definite analysis and assessment have been performed. In addition, the method by which thyroid hormone is measured has changed dramatically in recent years. Oppenheimer and coworkers originally succeeded in measuring free thyroid hormones in 1963 [7]; subsequently, various techniques and procedures have been provided to detect free thyroxine (FT4) and free tri-iodothyronine (FT3). In Japan in 1980s, most blood test results included measurements of total thyroxine (T4) and tri-iodothyronine (T3) binding with target binding proteins. Especially among malnourished patients, as well as CKD patients, a reduction in total binding protein led to reduction in total T4 and total T3; this might have caused erroneous diagnosis of hypothyroidism.

This study aimed to ascertain the frequency at which types of hypothyroidism were recognized in CKD patients, and to evaluate factors affecting thyroid function.

## Materials and methods

### Study design and setting

**Figure 1.**
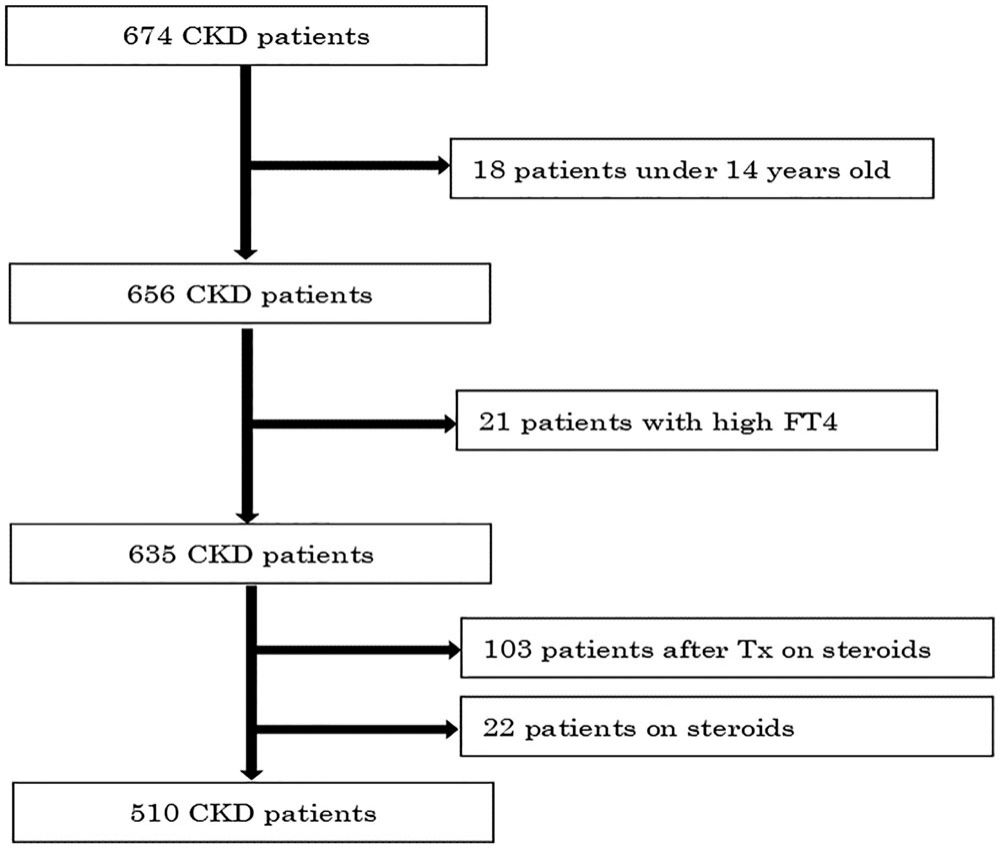
Algorithm for this study. CKD: chronic kidney disease; Tx: transplantation.

This cross-sectional study recruited Japanese CKD patients with suspected thyroid dysfunction, based on clinical symptoms as indefinite symptoms mentioned above at our outpatient clinic during the period from April 2012 through October 2016. Total number of CKD patients were 6633 in this period including dialysis patients. We did not exclude the patients receiving the thyroid hormone because they were not always euthyroid. Initially, 674 patients were accepted to measure the thyroid hormone and antithyroid antibodies (ATAb). We excluded under 15 years old (*n* = 18) and high free T4 (*n* = 21), caused by Graves’ disease. Additionally, we excluded patients receiving steroid treatment to avoid biased assessment of thyroid function. Of the remaining 635 patients, we excluded 103 for receiving steroid treatment after kidney transplant and 22 for receiving steroids for their original diseases (Figure 1). A total of 510 CKD patients was identified to this study.

### Laboratory measurements

Each patient was classified into five stages of CKD, on the basis of estimated glomerular filtration rate (eGFR) and amount of urinary protein (UP), in accordance with Japanese Society of Nephrology 2009 evidence-based practice guidelines for treatment of CKD [8]. Patients were examined in the laboratory in our hospital, and the following laboratory values were measured; eGFR, albumin (Alb), thyroid-stimulating hormone (TSH), FT3, FT4, and UP. Additionally, serum anti-thyroid peroxidase antibody (TPOAb) and anti-thyroglobulin antibody (TgAb) were measured. FT3, FT4, and TSH levels, as well as TPOAb and TgAb concentration were determined by electro-chemiluminescence immunoassay (ECLIA, Roche Diagnostics K.K., Tokyo, Japan). Reference ranges of FT3, FT4, TSH, TPOAb, and TgAb were 2.26–4.15 pg/ml, 1.01–1.67 ng/dl, 0.32–4.12 mIU/ml, <16 IU/ml, and <28 IU/ml, respectively.

### Assessment of kidney function and thyroid function

We determined Japanese eGFR as follows: (eGFR (ml/min/1.73 m^2^))=194 × Cr^−1.094^×Age^−0.287^ (×0.739 for female patients) [9]. CKD stage was classified in accordance with the Clinical Practice Guideline for Diagnosis and Treatment of CKD 2009 [8]. eGFR was divided into six categories as follows: G1, ≥90; G2, 60–89; G3a, 45–59; G3b, 30–44; G4, 15–29; and G5, <15 ml/min/1.73 m^2^. Proteinuria was divided into three categories as follows: A1, <0.15; A2, 0.15–0.49; and A3, ≥0.50 g/day [10]. The amount of UP per day was converted to g/gCr (grams per gram of creatinine).

Overt hypothyroidism (OH) was defined as low FT4 (<1.01 ng/dl) with elevated TSH (>4.12 mIU/ml). Subclinical hypothyroidism (SH) was defined as elevated TSH (>4.12 mIU/ml) with normal FT3 and FT4 (http://www.j-endo.jp/modules/patient/index.php?content_id=39, only in Japanese). Non-thyroidal illness (NTI) was defined as low FT3 with normal or low FT4 and TSH. Findings other than these three thyroid states were regarded as normal.

### Covariates

Covariates of age and gender were assessed based on self-reporting by each patient.

### Statistical analysis

Statistical analysis was performed using JMP 13 software (SAS Institute, Inc., Cary, NC). Continuous variables were expressed as median (range) and categorical variables were expressed as number (percentage). Statistical significance for the two groups was assessed using Student’s *t*-test for continuous variables, the Pearson *χ*^2^ test for categorical variables. We performed a univariate analysis to assess the statistical significance for each group using the Steel-Dwass test for non-parametric analysis. Nominal logistic regression analysis was used to perform a multivariate analysis. The analyzed values were expressed as adjusted odds ratio (OR) for the explanatory variables. We used Chi-squared test to determine the antibody positive ratio between OH and SH. Differences with *p*< .05 were considered statistically significant.

## Results

### Patients characteristics

Baseline demographic, clinical, and biochemical data of the 510 CKD patients are shown in Table 1. In all patients, age at recruitment was 67 (39–84) years and 230 patients (45.0%) were female. eGFR was 16.8 (4.3–73.37) ml/min/1.73 m^2^, UP was 1.8 (0.2–9) g/gCr, and Alb was 3.5 (2.3–4.5) g/dl. Each factor exhibited a non-normal distribution.

**Table 1.** Characteristics of individuals separated by thyroid function.

	Overt hypothyroidism *n* = 63, 12.0%	Subclinical hypothyroidism *n* = 76, 14.9%	NTI *n* = 169, 33.1%	Normal *n* = 202, 39.6%	All *n* = 510, 100%
Age, years Median, *n* (10–90%, percentile)	68 (47.6–85.6)*	70 (46.7–87)^#^	68.5 (41.2–84)^$^	61.5 (34.3–80)	67 (39–84)
Gender, *n* (%) Female	*n* = 30, 47.6*	*n* = 26, 34.2^#^	*n* = 83, 48.8^$^	*n* = 91, 45.1	*n* = 230, 45.0
eGFR, ml/min per 1.73 m^2^ Median, *n* (10–90%, percentile)	9.7 (4.02–57.92)*	13.5 (4.4–37.22)^#^	8.4 (3.78–42.84)^$^	43.8 (5.62–85.1)	16.8 (4.3–73.37)
Urine protein, g/gCr Median, *n* (10–90%, percentile)	5.1 (0.4–11.78)*	2.45 (0.2–10.66)^#^	2.2 (0.4–9.3)^$,^**	0.8 (0.2–4.94)	1.8 (0.2–2.9)
Albumin, g/dl median, *n* (10–90%, percentile)	2.8 (1.6–3.9)*	3.3 (2.4–4.23)^#^	3.2 (2.1–3.92)^$^	3.9 (3.1–4.58)	3.5 (2.3–4.3)

NTI: non-thyroidal illness; Cr: creatinine; dl: deciliter.

Values are median, quartile, number, and percentage.

* *p*<.05 normal vs. overt hypothyroidism.

# *p*<.05 normal vs. subclinical hypothyroidism.

$ *p*<.0001 normal vs. NTI.

** *p* < 0 .05 NTI vs. overt hypothyroidism.

**Figure 2.**
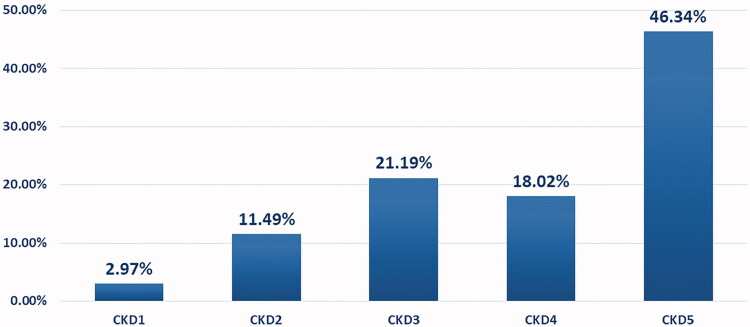
Distribution diagram of CKD stage.

The 510 patients were classified into five CKD stages (Figure 2) as follows: stage 5, 46.34%; stage 4, 18.02%; stage 3, 21.19%; stage 2, 11.49%; and stage 1, 2.97%. They were also classified into four groups by thyroid function, as follows: OH, 12.0%; SH, 14.9%; NTI, 33.1%; and normal, 39.6% (Table 1). In the univariate analysis, there were significant differences in age, eGFR, UP, and Alb among OH, SH, NTI, and normal groups, but there were no differences in gender.

**Figure 3.**
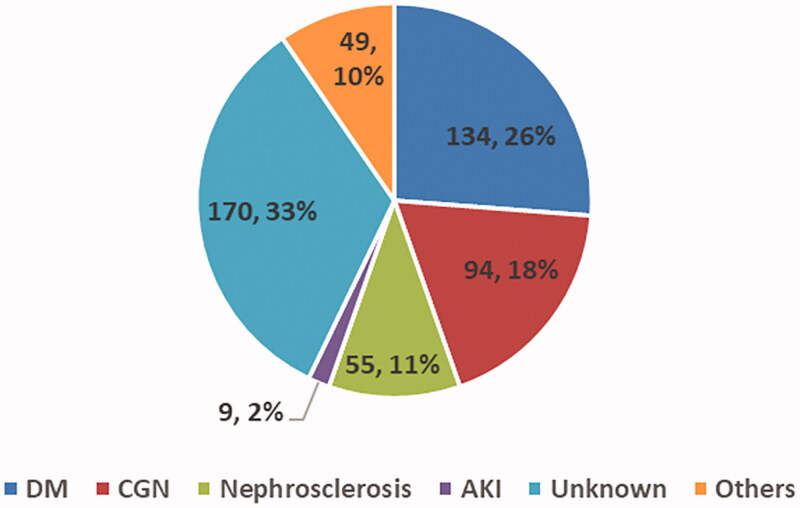
Pie chart of underlying disease in CKD patients. DM: diabetic mellitus; CGN: chronic glomerulonephritis; AKI: acute kidney injury.

Underlying diseases in CKD patients were as follows (Figure 3): diabetic nephropathy (*n* = 134, 26.0%), chronic glomerulonephritis (*n* = 78, 15.0%), nephrosclerosis (*n* = 68, 14.0%), acute kidney injury (*n* = 11, 2.0%), others (*n* = 47, 9.0%), and unknown (*n* = 172, 34.0%). AKI did not occur during CKD, but was referring to the transition of AKI-to-CKD. Twelve (2.4%) of 510 patients were diagnosed with nephrotic syndrome, based on the Japanese Society of Nephrology 2014 Evidence-Based Clinical Practice Guidelines for Nephrotic Syndrome [11].

### Association of overt hypothyroidism, subclinical hypothyroidism, and NTI with independent factors

The association of OH, SH, and NTI with independent factors is presented in Table 2. Since Alb level is strongly associated with the amount of the urine protein, we recognized this as intermediate variable (intermediate factor) and chose these four factors for the confounders: age, gender, eGFR, and urine protein. Age, eGFR, and UP were independently associated with OH, SH, and NTI in univariate and multivariate analyses but there were no gender differences. Urine protein had the highest OR of all factors.

**Table 2.** Independent factors associated with overt hypothyroidism, subclinical hypothyroidism, and NTI with CKD.

	Overt hypothyroidism			
	Univariate analysis		Multivariate analysis	
	OR (95%CI)	*p* Value	OR (95%CI)	*p* Value
Age, years	1.03 (1.01–1.05)	.017	1.04 (1.01–1.07)	.0039
Female gender	0.93 (0.53–1.65)	.8115	0.66 (0.29–1.48)	.317
eGFR, ml/min/1.73 m^2^	0.96 (0.94–0.97)	<.0001	0.96 (0.95–0.99)	.0013
Urine protein, g/gCr	1.34 (1.18–1.46)	<.0001	1.31 (1.16–1.48)	<.0001
	Subclinical hypothyroidism			
	Univariate analysis		Multivariate analysis	
	OR (95%CI)	*p* Value	OR (95%CI)	*p* Value
Age, years	1.04 (1.02–1.05)	<.0001	1.05 (1.02–1.08)	.0005
Female gender	1.69 (0.97–2.06)	.0654	1.32 (0.61–2.87)	.4764
eGFR, ml/min/1.73 m^2^	0.95 (0.94–0.97)	<.0001	0.96 (0.94–0.98)	.0003
Urine protein, g/gCr	1.19 (1.08–1.30)	.0002	1.18 (1.06–1.32)	.0027
	NTI			
	Univariate analysis		Multivariate analysis	
	OR (95%CI)	*p* Value	OR (95%CI)	*p* Value
Age, years	1.02 (1.01–1.04)	<.0001	1.03 (1.01–1.04)	.0068
Female gender	0.89 (0.59–1.35)	.588	1.01 (0.57–1.78)	.9772
eGFR, ml/min/1.73 m^2^	0.97 (0.96–0.97)	<.0001	0.97 (0.96–0.99)	.0047
Urine protein, g/gCr	1.20 (1.10–1.31)	<.0001	1.14 (1.04–1.24)	<.0001

OR: odds ratio; CI: confidence interval; eGFR: estimated glomerular filtration ratio; CKD: chronic kidney disease.

Age, eGFR, and UP were independently associated with OH, SH, and NTI in univariate and multivariate analyses but there were no gender differences. Urine protein had the highest OR of all factors.

### Frequency distribution

**Figure 4.**
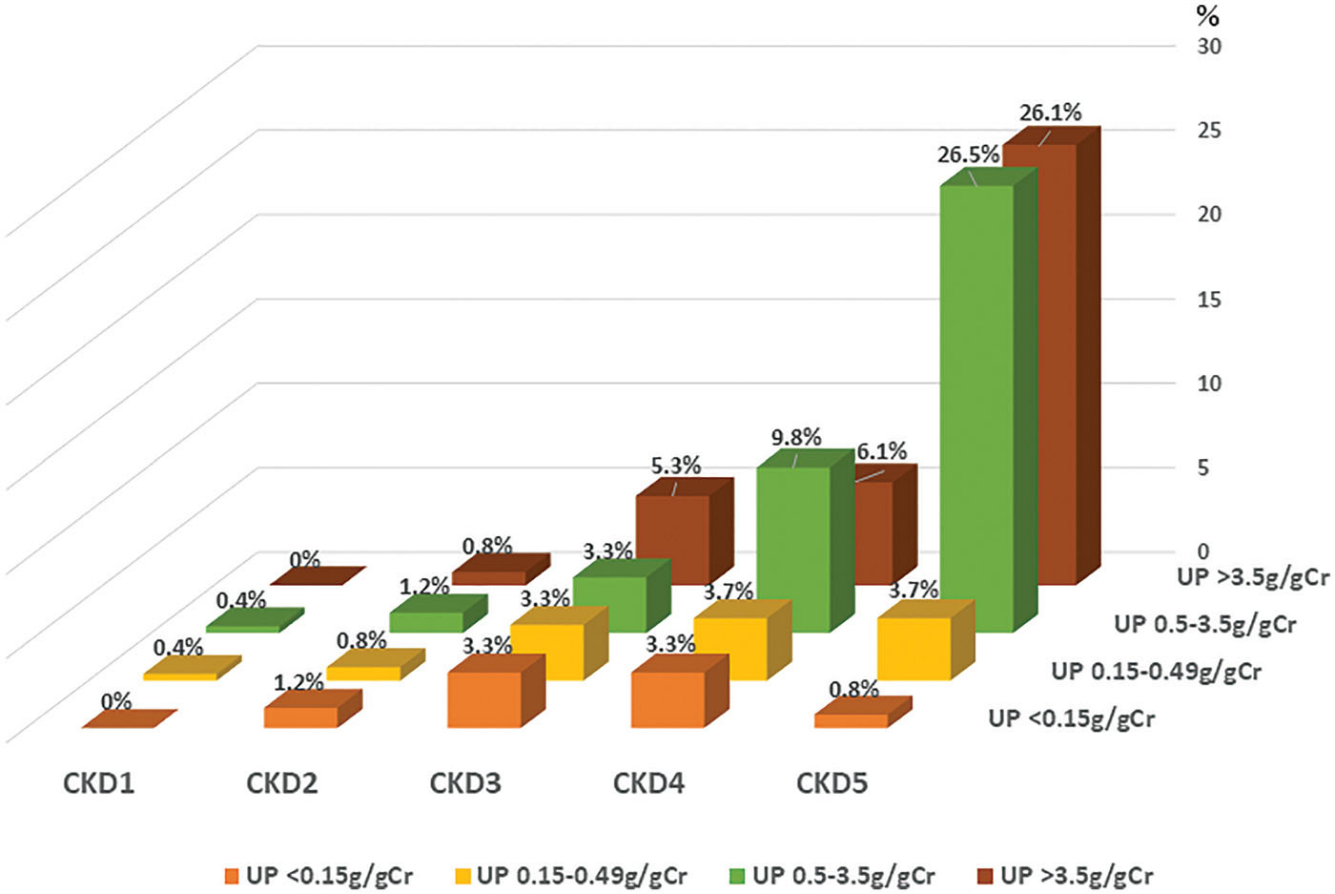
Association of hypothyroidism; overt, subclinical, and NTI with CKD stage and amount of urinary protein. UP: urinary protein.

Frequency distributions showing associations of hypothyroidism (OH, SH, and NTI) with CKD stage and quantity of UP are depicted in Figure 4. Out of 308 subjects of the thyroid dysfunction group (OH, SH, and NTI), three subjects had no data of eGFR. In addition, 60 subjects had no data of urine protein because of oliguria. Totally, 245 subjects were analyzed with CKD stage and amount of urine protein. This analysis revealed that the prevalence of thyroid dysfunction was greater among more severe stage of CKD with the higher amount of UP: CKD 5 with higher amount of UP showed 26.5–26.1% of thyroid dysfunction group.

### Antithyroid antibodies; anti-thyroglobulin antibody and anti-thyroid peroxidase antibody

Of the 131 patients who underwent measurement of TgAb, 32 exhibited positive antibody (≧28, 24.4%). Likewise, 125 underwent measurement of TPOAb, of which 35 exhibited positive antibody (≧16, 28.0%). Table 3 shows the number and percentage of TgAb and TPOAb in OH, SH, and NTI plus normal subjects. 37.5% of OH showed positive TgAb and 40.0% of OH showed positive TPOAb. 12.5% of SH showed positive TgAb and 17.1% of SH showed positive TPOAb. On the other hand, in NTI plus normal group, 50.0% positivity of TgAb and 42.9% positivity of TPOAb were observed.

**Table 3.** TgAb and TPOAb of overt hypothyroidism, subclinical hypothyroidism, and NTI plus normal subjects.

	Total, *n*, %	OH, *n*, %	SH, *n*, %	NTI + normal, *n*, %
TgAb positive	32, 24.4%	12, 37.5%	4, 12.5%	16, 50.0%
TgAb negative	99, 75.6%	23, 23.23%	24, 24.24%	52, 52.53%
TPOAb positive	35, 28.0%	14, 40.0%	6, 17.1%	15, 42.9%
TPOAb negative	90, 72.0%	18, 20.0%	22, 24.4%	50, 55.6%

TgAb: anti-thyroglobulin antibody; TPOAb: anti-thyroid peroxidase antibody; OH: overt hypothyroidism; SH: subclinical hypothyroidism.

TgAb ≧ 16, TPOAb ≧ 28: antibody-positive, values are number and percentage.

## Discussion

This study revealed a high prevalence of hypothyroidism among CKD patients, such that there was a significant association between hypothyroidism and the presence of CKD. Furthermore, age, eGFR, amount of UP, and Alb level were important factors influencing the prevalence of hypothyroidism. However, gender was not associated with hypothyroidism among CKD patients unlikely to general population. Additionally, our frequency distribution analysis showed that the prevalence of OH and SH exhibited upward trends at higher stages of CKD, especially when the amount of UP increased. Third, it was difficult to diagnose NTI, OH, and SH among CKD patients because symptoms of CKD overlapped with those related to conditions of thyroid dysfunction.

Over the past few decades, many articles have been published regarding interactions between thyroid function and kidney function [2,3,5,12,13]. Hypothyroidism can induce kidney dysfunction by reducing renal blood flow and glomerular filtration rate [14], and by inducing tubular dysfunction [15]. Furthermore, in CKD patients with hypothyroidism, thyroid hormone replacement therapy (THRT) may attenuate the reduction of renal function [16]. CKD has also been suggested to affect thyroid function; continuous kidney dysfunction may contribute to the onset of hypothyroidism and/or NTI [4,5,17,18], but the details are not yet clear. There are also several factors to affect the association between CKD and thyroid function including the dysfunction of renal excretion due to CKD. It may decrease the clearance of inorganic iodine and excessive iodine may reduce the synthesis of thyroid hormone by the Wolff–Chaikoff effect [15]. Chronic acidemia and malnutrition also may inhibit deiodination from T4 to T3 in the peripheral tissues.

In the general population, the prevalence of hypothyroidism is 4.6% in the United States, but some differences exist among ethnicities [19]. The prevalence of hypothyroidism is 0.7–2.1% in Japan [20], but there are no official population-based data regarding the incidence of hypothyroidism among Japanese adults. In Korea, the prevalence of OH and SH is 0.73% (males, 0.40%; females, 1.10%) and 3.10% (males, 2.26%; females, 4.04%), respectively [21]. In the present study, the prevalence of OH was 12.0%, while that of SH was 14.9%. These were higher than those in previous reports, but showed no gender difference. It is an established theory that there is a greater incidence of hypothyroidism among females [19–21]. However, our data showed a notable result in thyroidology to find no female predominance, which reflected that hypothyroidism derived from CKD rather than gender difference. In general, several factors influence thyroid function. For example, TSH reportedly increases with age [22,23]. However, the present study revealed no age-related changes in TSH (data not shown). Furthermore, our results revealed a relationship between UP and hypothyroidism. UP had the highest OR of all the factors analyzed, indicating that it is a strong predictor of hypothyroidism. Although we revealed a relationship between UP and hypothyroidism, there was not a direct relationship between UP and eGFR. Frequency distribution analysis also showed that hypothyroidism increased with higher amounts of UP and that this effect was greater at higher stages of CKD. Notably, more than 99% of T3 and T4 are bound to proteins. Typically, they bind to thyroxine-binding globulin; smaller proportions of the remaining T3 and T4 are bound to other proteins, such as transthyretin, as well as preAlb and Alb in blood [24]. In children, congenital nephrotic syndrome involves the leakage of thyroid-hormone bound proteins from the blood into the urine; thus, THRT is necessary for affected children [25–27]. In adults, this mechanism has not yet been clarified, but may be similar. In specific types of CKD as minimal change nephrotic syndrome, focal segmental glomerulonephritis and membranous nephropathy, much proteinuria is often present, such that thyroid hormones might leak into the urine. To clarify the association between UP and hypothyroidism, further investigation is needed.

In CKD patients, low Alb indicates malnutrition. Dietary recommendations in end-stage renal disease to prevent malnutrition are difficult, because increases in protein intake generally result in concomitant increases in serum potassium or phosphorus [28]. This dietary conflict may cause patients to be undernourished. Notably, our results suggest that malnutrition related to low Alb due to proteinuria might be associated with hypothyroidism.

Moreover, in the general population, most patients with hypothyroidism exhibited chronic thyroiditis caused by Hashimoto’s thyroiditis with positive ATAb [20,29]. However, in the present study, patients with OH had 37.5% positive in TgAb and 40.0% in TPOAb. SH had 12.5% positive in TgAb and 17.1% positive in TPOAb (Table 3). NTI plus normal group showed higher positivity of TgAb and TPOAb. As the number of this subject in this table was very small, we could not analyze statistically, but these data may suggest that hypothyroidism complicated by CKD may involve different onset mechanisms from those occurring in the general population.

Not a few clinicians have not recognized hidden hypothyroidism in CKD patients and elements of thyroid dysfunction may be misdiagnosed as NTI. In fact, many patients with critical illness or chronic illness (e.g., CKD) are likely to exhibit low T3 and if their condition worsens, they may also exhibit low T4 [30]. This manifestation should be diagnosed as NTI. In this situation of NTI, although the data may show a low level of thyroid hormones, clinically the patient is not hypothyroid but euthyroid. Thus, THRT may not be appropriate for these patients, while treatment of underlying disease may be beneficial [31]. Therefore, thyroid dysfunction should be carefully distinguished in order to ensure appropriate treatment in CKD patients.

CKD as chronic illness may be complicated with NTI [18,30,32,33]. Suppression of the hypothalamus-pituitary-thyroid (HPT) axis, reduction of T4 conversion to T3 with increased plasma rT3, and reduction in T3 demand in the peripheral organs [32] might occur as a biological reaction. However, several papers investigating NTI reported that rT3 was not increased in kidney dysfunction [32,34]. This inconsistency is controversial. Furthermore, we could not technically order the measurement of rT3 in general at our hospital and we had no data. Nevertheless, the thyroid function of NTI is quite different from OH and SH, and there is no evidence-based consensus or guideline for treatment of NTI with thyroid hormone [30,33,35]. Hence, we designated NTI as a separate condition from the other two types of hypothyroidism in the present study. THRT is clearly needed for OH, and moreover, there have been various reports on the use of THRT for SH. Shin et al. reported that THRT for SH could inhibit progression of CKD (the inverse relationship was also observed) [16]. However, Kaptein et al. reported that, in CKD, isolated TSH elevation did not indicate mild thyroid gland failure [36] and that the existence of SH might be uncertain. Indeed, multiple medical institutions have used THRT for empirical treatment of patients with SH.

As mentioned previously, various factors affect thyroid function, and the use of some drugs may contribute. In 2012, Nishikawa provided an official report indicating that severe side effects of some drugs could cause thyrotoxicosis and hypothyroidism [37]. Notably, various types of drugs are causative agents, including steroids, which suppress TSH production and secretion. Fortunately, most such medications do not cause clinically distinct central hypothyroidism [38]. In clinical settings, many CKD patients require steroid therapy. For the present study, we specifically selected patients without steroid therapy. TSH level is important for clarifying the type of hypothyroidism. Steroid administration might cause SH to mimic normal through suppression of TSH (high to normal, with normal T3 and T4), and cause OH to mimic NTI through suppression of TSH (high to normal, with low T3 and/or low T4).

The present study had several limitations. First, we did not perform thyroid function tests for all patients at the outpatient clinic of the kidney center (*n* = 6633); we checked thyroid function only for patients with accepted to measure thyroid dysfunction (*n* = 674). Second, we did not measure ATAb in all patients; we checked these antibodies only for patients with suspected autoimmune thyroid disease. Third, we did not measure the concentration of rT3. In Japan, we would rarely measure rT3 in these days and we could not technically order the measurement of rT3 in general at our hospital. In addition, several papers reported that ‘rT3 was not increased in kidney dysfunction’ [32,34], and thus could not definitively diagnose NTI. Fourth, we did not prescribe THRT to all OH and SH patients in order to evaluate the treatment outcome.

In conclusion, hypothyroidism complicated by CKD showed clear associations with age, eGFR, Alb and most notably, the amount of UP. To further establish the relationship between thyroid function and UP, additional studies are needed with a focus on protein leakage into the urine. Finally, because hypothyroidism complicated by CKD has a variable and complex background, the assessment of thyroid function is challenging and requires careful analysis for precise diagnosis, and treatment.
